# Lateral Flow Biosensor for On-Site Multiplex Detection of Viruses Based on One-Step Reverse Transcription and Strand Displacement Amplification

**DOI:** 10.3390/bios14020103

**Published:** 2024-02-17

**Authors:** Xuewen Lu, Kangning Ding, Zhiyuan Fang, Yilei Liu, Tianxing Ji, Jian Sun, Zhenling Zeng, Limin He

**Affiliations:** 1 National Risk Assessment Laboratory for Antimicrobial Resistance of Animal Original Bacteria, South China Agricultural University, Guangzhou 510642, China; awen5983@163.com (X.L.); kangningd1997@163.com (K.D.); zlzeng@scau.edu.cn (Z.Z.); 2 School of Biomedical and Pharmaceutical Sciences, Guangdong University of Technology, Guangzhou 510006, China; fangjnu@126.com; 3 Guangdong Provincial Key Laboratory of Veterinary Pharmaceutics Development and Safety Evaluation, College of Veterinary Medicine, South China Agricultural University, Guangzhou 510642, China; yileiliu19@163.com; 4 Clinical Laboratory Medicine, The Second Affiliated Hospital of Guangzhou Medical University, Guangzhou 510260, China; jitianxing7021@163.com; 5 National Reference Laboratory of Veterinary Drug Residues (SCAU), College of Veterinary Medicine, South China Agricultural University, Guangzhou 510642, China

**Keywords:** lateral flow biosensor, RT-SDA, AuNPs, on-site detection, NAATs

## Abstract

Respiratory pathogens pose a huge threat to public health, especially the highly mutant RNA viruses. Therefore, reliable, on-site, rapid diagnosis of such pathogens is an urgent need. Traditional assays such as nucleic acid amplification tests (NAATs) have good sensitivity and specificity, but these assays require complex sample pre-treatment and a long test time. Herein, we present an on-site biosensor for rapid and multiplex detection of RNA pathogens. Samples with viruses are first lysed in a lysis buffer containing carrier RNA to release the target RNAs. Then, the lysate is used for amplification by one-step reverse transcription and single-direction isothermal strand displacement amplification (SDA). The yield single-strand DNAs (ssDNAs) are visually detected by a lateral flow biosensor. With a secondary signal amplification system, as low as 20 copies/μL of virus can be detected in this study. This assay avoids the process of nucleic acid purification, making it equipment-independent and easier to operate, so it is more suitable for on-site molecular diagnostic applications.

## 1. Introduction

Acute respiratory illnesses (ARIs) caused by respiratory viruses affect hundreds of millions of people per year [1,2,3], especially RNA viruses, whose spreading is difficult to mitigate due to the high rate of mutation [4,5]. ARIs had great impact on public health in previous outbreaks. According to the WHO statistics, the COVID-19 outbreak in 2019 has caused over 770 million infections and nearly 7 million deaths (https://covid19.who.int/, accessed on 5 October 2023). Influenza, as a globally monitored disease, causes approximately 1 billion infections and 290–650 thousand deaths annually (https://www.who.int/news-room/fact-sheets/detail/influenza, accessed on 5 October 2023). Multiple respiratory pathogen infections often break out in the same epidemic season with similar symptoms, such as coughing, runny nose, fever, et cetera [6,7,8]. Therefore, accurate and fast on-site testing kits that could speed up the identification of viral pathogens play an important role in blocking the spread of epidemics. The most extensively used tools are rapid antigen detection tests, owing to their ease of use, low cost, and short detection time [9,10,11]. However, many reports have stressed the high rate of false results of these assays [12,13,14]. Nucleic acid amplification tests overcome the shortcomings of antigen detection methods due to their high sensitivity and specificity [15,16]. Commonly, lab-based multiplex PCR systems can detect many respiratory viruses in one test [17,18,19], but these assays require highly trained operators to handle the specimens and nucleic acid with specialized equipment, which hinders the popularization of such methods in point-of-care testing (POCT) scenarios [20]. In comparison to PCRs, isothermal nucleic acid amplification tests (e.g., NASBA, LAMP, RCA, RPA) are faster, and can be carried out at a constant temperature, avoiding the need of sophisticated equipment like thermocyclers, thus greatly improving the chance for on-site use [21,22,23,24,25]. But, even after optimization, isothermal nucleic acid amplification tests cannot detect single-nucleotide polymorphisms (SNPs), which could be crucial in both pathogen and disease detection. Clustered regularly interspaced short palindromic repeats (CRISPR)-associated nuclease (Cas)-based systems could identify nucleic acid sequences more accurately, even for single-nucleotide polymorphisms (SNPs). Due to their selectivity and programmability, CRISPR-Cas systems have been introduced into isothermal amplification assays to improve specificity in recent years [26,27], such as the detection of SARS-CoV-2 and influenza A and B from respiratory swab RNA extracts [28,29].

**Figure 1:**
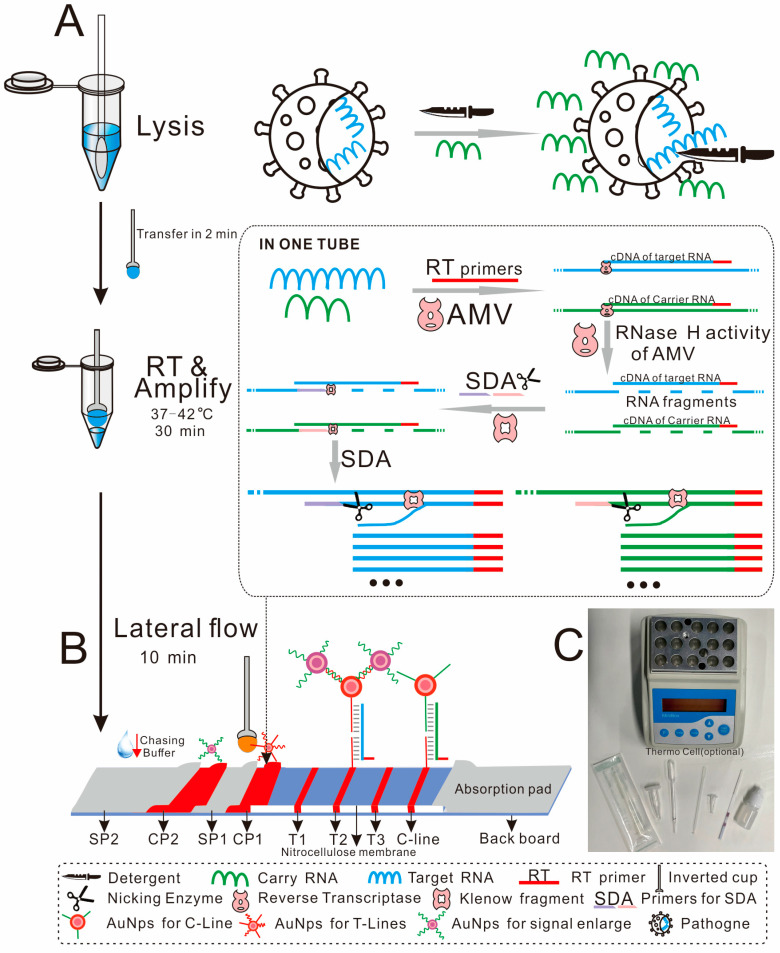
Principle and workflow of this detection. (**A**) Specimen was lysed by detergent in the lysis buffer. The yield buffer was directly transferred to the enzyme mixture for SDA amplification. (**B**) One inverted cup of the amplified product was loaded on the CP1, and 3 drops of chasing buffer were loaded on the SP2 for visual detection. (**C**) All the materials used in this assay. The lower part of the photo, from left to right are swab, lysis buffer, dropper, inverted cup, enzyme mixture, strip and chasing buffer.

All the nucleic acid amplification tests mentioned above require tedious DNA/RNA extraction, which greatly limits their use in the point-of-care testing field. Microfluidic technology has advantages in nucleic acid extraction, but this platform still requires specialized equipment [30,31]. The direct PCR method avoids a tedious DNA extraction process, but it is only suitable for some specific samples and requires specialized equipment [32]. So far, there is no RNA extraction-free biosensor that does not require equipment. Almost all the developed nucleic acid strips detect their targets by converting nucleic acid signals into immune signals, such as the binding of small molecules, antibodies or streptavidin and biotin [27], making it difficult to achieve high-throughput and high-specificity detection. The strips that display results through nucleic acid hybridization have higher throughput and better specificity compared to immunological methods. In our previous works, we have developed an aptamer-based DNA-extraction-free biosensor for the detection of salmonella and *Escherichia coli* O157:H7 [33,34]. The biosensor avoided the tedious nucleic acid extraction process. However, it was an aptamer-dependent system that was unable to detect its target without an aptamer, and several rounds of washing were needed before amplification. To further resolved this problem, in this study, we developed an extraction-independent on-site biosensor for rapid and multiplex detection of single-stranded RNAs (ssRNAs) from viruses. Samples were first lysed in lysis buffer to release the target ssRNAs. Then, the target RNAs were reverse transcribed into cDNAs, and further amplified by SDA. The yield ssDNAs were visually detected by a lateral flow biosensor (Figure 1). As low as 20 copies/μL of viruses were detected.

## 2. Materials and Methods

### 2.1. Reagents and Chemicals

Oligonucleotides, deoxynucleoside triphosphates (dNTPs) and AMV Reverse Transcriptase were purchased from Shanghai Sangon Biological Engineering Technology (Shanghai, China). Klenow Fragment (3′→5′ exo-) (M0212) and Nt.AlwI (R0513) were purchased from New England biolabs (Ipswich, MA, USA). HAuCl_4_ was purchased from Sigma-Aldrich (Steinheim, Germany). Bovine Serum Albumin (BSA) was supplied by Proliant New Zealand limited (Feilding, New Zealand). Nitrocellulose membrane was purchased from Sartorius (Goettingen, Germany). PVC back board, fiberglass and absorbent paper were purchased from Shanghai Kinbio (Shanghai, China). Streptavidin (SA) was purchased from Wuhan Aokebotai Biotechnology Co., Ltd. (Wuhan, China). 2 × Taq plus PCR Master Mix (with red dye) was purchased from biosharp life science (Hefei, China). Carrier RNA was extracted from the leaf of rice (*Oryza sativa* L.) by Trizol^TM^ (15596026) in our lab. Trizol^TM^ was purchased from Thermo Fisher Scientific (Waltham, MA, USA). All the solutions used in this study were prepared in our lab. All the chemicals used in this study were purchased from standard commercial sources and were of analytical-grade purity.

### 2.2. Preparation of Gold Nanoparticles (AuNPs)–Oligo Conjugates

AuNPs with an average diameter of 25 nm were prepared according to the following protocol. Briefly, 12.5 mL of 2% (*w*/*v*) trisodium citrate was quickly added into 500 mL of a rapidly stirred and boiled HAuCl_4_ solution (0.04%) in a 1000 mL conical flask. After turning red (about 60 s), the solution was boiled for an additional 5 min. Then, the solution was cooled to room temperature with gentle stirring. The AuNPs solution was stored at 4 °C until use.

To prepare AuNPs–oligo conjugates, 12 μL of 0.2 M K_2_CO_3_ solution was added into 1 mL of the prepared AuNPs solution. The solution was mixed by inverting the tube 10 times, followed by adding 20 μg Streptavidin into the mixture, and shaking it for 5 min. After that, 20 μL of 10% BSA solution (diluted in ultral pure water) was added into the mixture. Four microliters of biotin-modified oligo (100 μM, diluted in 20 mM PBS containing 3 mM EDTA) was added in the previously prepared solution and shaken for 10 min. AuNPs–oligo conjugates were collected by centrifugation at 6 × 10^3^× *g*, for 15 min at 4 °C, and re-suspended in 100 μL of resuspending buffer (30 mM Tris, 0.1% PVP-40, 0.6% Casein, 0.2% BSA, 0.3% trisodium citrate, 3 mM EDTA, 2% sucrose, 0.06% Triton X-100, 0.06% Tween-20, 0.05% NaN_3_, pH = 7.5). AuNPs–oligo conjugates were dispensed onto a fiberglass membrane and dried as a conjugation pad.

### 2.3. Construction of Lateral Flow Biosensor

All the capture probes for T-lines and C-line were diluted to 50 mM in dispensing buffer (20 mM PBS, 0.15 mg/mL BSA, 1 mM EDTA). The solutions were dispensed onto the nitrocellulose membrane at a speed of 0.7 μL/cm by a lateral flow dispenser (Shanghai kinbio, Shanghai, China). The membrane was treated with ultraviolet light for 15 min and then dried at 42 °C for 15 h. Fiberglass membrane was used as sample pad after being treated with sample pad buffer (0.3% Tween-20, 0.1% Triton X-100, 0.3% BSA, 3 mM EDTA). It was dried and stored at low humidity (RH < 30%) at room temperature. Conjugation pad1 (CP1) was sprayed with gold nanoparticles (AuNPs) specific for the T-lines and C-line; the gold nanoparticles (AuNPs) specific for the T-line were co-modified with En-prob1 and one kind of Au probe specific for the corresponding T-line. Conjugation pad2 (CP2) was sprayed with gold nanoparticles (AuNPs) modified with En-prob 2, which can bind to the AuNPs captured by the T-lines to amplify the signal. The sample pad1 (SP1, about 12 mm width) can separate CP1 and CP2, allowing the two kinds of conjugations to pass through T-lines successively, making the chromatography of AuNPs smoother and reducing steric hindrance caused by the binding of signal amplification AuNPs. So, the SP1 could improve capture efficiency of T-line for the gold nanoparticles on CP1.The sample pad2 (SP2) was used for chasing buffer loading and releasing. The components of SP1 and SP2 are the same, except for differences in their width and position on the test strip.

Sample pad2 (SP2), conjugation pad 2 (CP2), sample pad1 (SP1), conjugation pad 1 (CP1), nitrocellulose membrane and the absorbent pad were attached along the long axis of an adhesive back board with an overlap of 1–2 mm and cut into 3 mm wide strips using a strip cutter (Shanghai kinbio, Shanghai, China) (Figure 1B).

### 2.4. Experimental Design

The inactivated cultured influenza A virus (IVA, H1N1) and influenza B virus (IVB) were supplied by Fapon Biological Company (Shenzhen, China). Chlamydia pneumoniae (TWAR Strain Cat.R02620) was supplied by Meridian life science (Memphis, TN, USA). *Escherichia coli* (*DH5*α), and yeast was obtained from C-king Biocompany (Guangzhou, China). Mycoplasma pneumoniae, RSV- and SARS-CoV-2-positive swabs were eluted by saline, and the swab eluent was inactivated after being treated at 60 °C for 30 min. All the swab eluent with known Ct value was obtained from the Second Affiliated Hospital of Guangzhou Medical University. All the procedures were approved by the Ethics Committee of The Second Affiliated Hospital of Guangzhou Medical University.

The samples were lysed by the lysis buffer (0.2% triton X-100, 0.5% NaCl, 0.2 μg/μL Carrier RNA) to release RNAs. Carrier RNA can slow down the degradation of target RNAs. The yielded solution was then transferred to an enzyme mixture, where the AMV enzyme synthesized the cDNA of the target region and removed the RNA from the cDNA/RNA complex, which created the binding sites for SDA primers; the resulting single-stranded cDNA was then used as a template for single-direction isothermal strand displacement amplification. The yielded ssDNA was further detected by a lateral flow biosensor (Figure 1). The added carrier RNA was also amplified by the same enzyme mixture with different primers as a quality control, and the yielded ssDNA of carrier RNA was captured by the probes in C-line. Thus, a convenient on-site diagnostic biosensor for ssRNA of pathogens was established.

### 2.5. Carrier RNA Preparation

Rice leaves were ground with liquid nitrogen, and about 100 mg of grounded leaves was transferred into a centrifuge tube with 1 mL of Trizol. The solution was well mixed. RNA was extracted following the instructions of the RNA extraction procedure. The purified RNA was dissolved in RNase-free water and fragmented with Ultrasonic Crusher (Ningbo Scientz Biotechnology, Ningbo, China) (180 W, 2 S, ON; 8S, OFF; 10 Cycles). It was stored at −80 °C for later use.

### 2.6. Detection of Pathogens

For the SDA amplification, 20 μL of the lysis mixture was added into 20 μL of the 2 × enzyme mixture (1 μM of each RT primer and 1 μM of each SDA primer, 10 U Nt.AlwI, 5 U AMV Reverse Transcriptase, 5 U Klenow Fragment (3′→5′ exo-), 50 mΜ dNTPs, 0.2 μg/μL Carrier RNA, 4 μL NEBuffer^TM^2 (10×) buffer and 4 μL AMV Reverse Transcriptase buffer (5×)). It was incubated at 37–42 °C for 30 min. After the amplification, about 10 μL of the yielded solution was transferred to the CP1 of the strip by an inverted cup, and 3 drops (about 80 μL) of chasing buffer were added (6 × SSC: 0.9 M NaCl, 0.09 M Sodium Citrate, pH 7.0). The result was observed by naked eye after 10 min.

The performance of this biosensor was also evaluated using spiked specimens. The nasal swabs’ elutions without the three target viruses (confirmed by PCR) were lysed with lysis buffer (without carrier RNA), and the obtained solution was used as matrix. Then, 20 μL of gradient-diluted virus sample or saline containing 0.8 μg/μL Carrier RNA was added into 60 μL of matrix. Then, the mixture was divided into two parts. One part was tested by this biosensor, and the other part was tested by RT-PCR.

Biosensor detection was carried out as follows. Then, 20 μL yield lysis buffer was transferred to 20 μL 2 × enzyme mixture. The solution was mixed several times and kept at 40 °C for 30 min. After amplification, one inverted cup (about 10 μL) of the mixture was loaded onto the CP1 of the biosensor, and 3 drops (about 80 μL) of chasing buffer were loaded onto the SP2.

To evaluate the stability of the target RNA in the lysis buffer in spiked specimen detection, the lysis buffer was transferred to the enzyme mixture for amplification at 1 min, 2 min, 5 min, 8 min, 10 min and 15 min after lysis. The amplified DNA was tested according to the previous procedure to seek the optimal lysis time for this assay.

For the RT-PCR test, 20 μL of the yield mixture was extracted by Trizol. The yielded RNA was dissolved in 20 μL RNase-free water, and 10 μL of the RNA was used for reverse transcription solution (containing 1 μM A-R primer, 20 U AMV, 4 μL AMV RT buffer (5×), 1 μL 25 mM dNTPs, and the reaction volume was adjusted to 20 μL with DEPC-treated water). The mixture was incubated at 42 °C for 30 min for reverse transcription. An amount of 5 μL of yield cDNA was transferred to 10 μL 2 × Taq plus mastmix containing 1 μM A-F and 1 μM A-R primers, and the reaction volume was adjusted to 20 μL with double-distilled water. The PCR was carried out as follows: 94 °C for 3 min, followed by 35 cycles of 94 °C for 30 s, 56 °C for 30 s, 72 °C for 10 s, followed by 72 °C for 3 min. The PCR products were run on 2% agarose gel (with 6 μL/100 mL 10,000 × SYBR green) in 0.5 × TBE (5.4 g/L Tris, 2.75 g/L Boric Acid, 1 mM EDTA, pH 8.0). As the length of the RT-PCR product was close to the 5S RNA of the added carrier RNA, a final concentration of 10 μg/mL of RNase A was added to the PCR product and incubated at 37 °C for 30 min to eliminate the interference of RNA before electrophoresis.

### 2.7. Ready-to-Use Kit Preparation

The SDA amplification mixture contains primers for RT and SDA, AMV enzymes, amplification enzymes, nicking enzyme and buffers, All of these components need to be prepared precisely in one tube for on-site testing. Therefore, we made a pre-made ready-to-use reaction tube according to the following procedure. Briefly, 20 μL of the 2 × enzyme mixture (please refer to Section 2.6 of this article for details) containing 5% (*w*/*v*) mannitol was transferred to a reaction tube and freeze-dried by a freeze dryer (SCIENTZ–30F, Ningbo Scientz Biotechnology, Ningbo, China) according to the following steps: −50 °C for 5 h, −40 °C for 2 h, −30 °C for 2 h, −20 °C for 2 h, −10 °C for 2 h, 0 °C for 2 h, 10 °C for 4 h, 20 °C for 1 h. The freeze-dried reaction tubes were stored in a self-sealing bag with desiccants until use. To evaluate the stability of this kit, the freeze-dried amplification tubes and 2 × enzyme mixture (containing mannitol) were stored at 45 °C in an oven for aging tests. Then, the aging reagents (oven treated) were tested in parallel with the freshly made enzyme mixture (without mannitol).

## 3. Results

### 3.1. Design of Probes for Pathogens Detection

**Figure 2:**
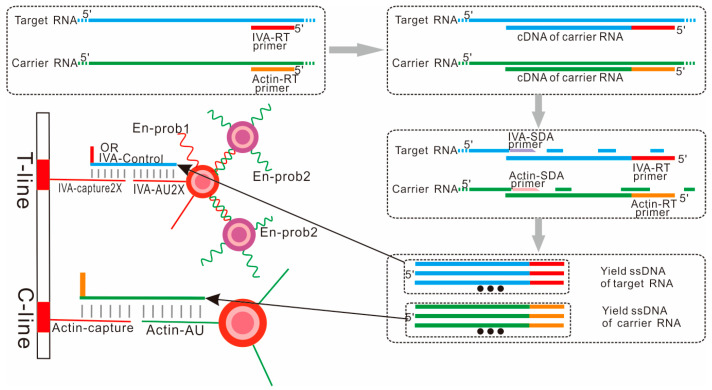
Demonstrates the relationship between different oligonucleotides using influenza A virus detection as an example.

The primers and probes for influenza A virus, influenza B virus, SARS-CoV-2 and β-actin of rice are shown in Table 1. The RT primers that trigger the reverse transcription process are complementary to the corresponding target RNAs. The SDA primers are complementary to the generated cDNAs. The capture probs and the AU probes are complementary to two ends of the yielded ssDNA, respectively. All of the AU probes are modified with a biotin at 3′ or 5′ ends for the conjugation of AuNPs, and the capture probes are coated on a nitrocellulose membrane to form T-lines and C-line, thus forming a sandwich-like link between the coating lines and AuNPs. The IVA-Control is synthesized oligonucleotide for quality control of the lateral biosensor, as the sequence of this oligo is the same as the amplification product of the IVA-positive sample. En-prob 1 and En-prob 2 are two kinds of probes for the signal amplification system; the sequence of En-prob 1 is complementary to the sequence of En-prob 2. En-prob1 and one kind of Au-probe specific for T-line are co-modified onto AuNPs for detecting corresponding target nucleic acids, while EN-prob 2 modified AuNPs can bind to the EN-prob 1 modified AuNPs for signal amplification. The sequences of some probes are doubled to improve the hybridization efficiency with target ssDNA. The relationships between all probes are shown in Figure 2.

### 3.2. Establishment of the Biosensor

**Figure 3:**
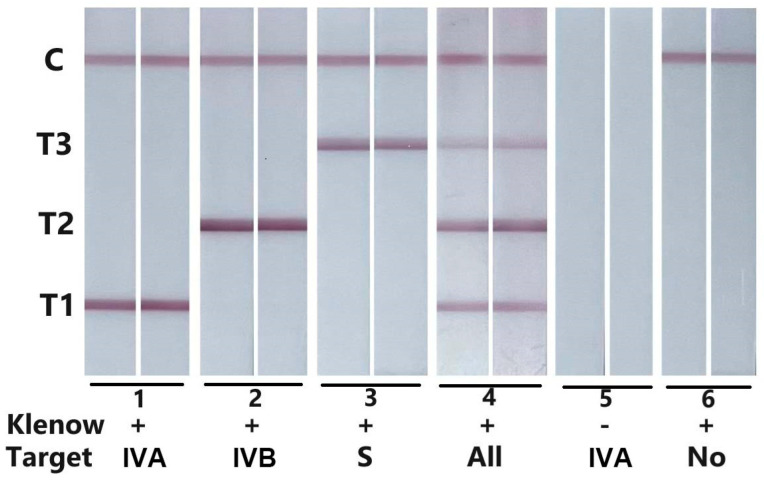
Typical images of the biosensor for viruses detection with different conditions. The capital letter IVA, IVB and S represent influenza virus A-positive samples, influenza virus B-positive samples and SARS-CoV-2-positive samples, respectively. “All” represents the existence of all three kinds of viruses. “No” represents the absence of all three kinds of viruses.

Primary experiments were carried out using inactivated virus or swab eluent. Figure 3 shows typical photos of the results. Red test lines are observed in the presence of the corresponding virus. No test line is observed in the absence of the target virus, and no line is observed in the absence of enzymes. All the reagents and probes used in the enzyme mixture do not affect the specificity of these biosensors. The results show that this biosensor worked well in ssRNA detection.

### 3.3. Optimization of the Biosensor

**Figure 4:**
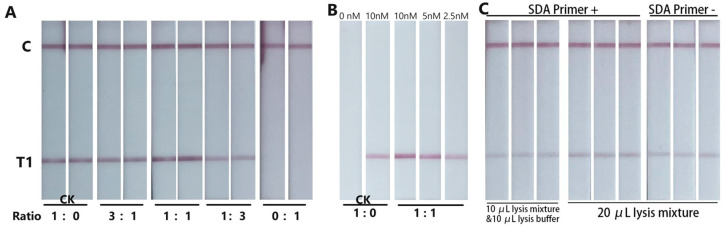
(**A**) Optimization of the biosensor with different ratio of specific AU probes and EN-prob1. (**B**) The signal of T-Line of different concentrations of A-prod. (**C**) Typical images of the biosensor with or without A-SDA primer.

In order to improve the sensitivity of the biosensor, a signal amplification system was introduced. The AuNPs dispensed on the conjugation pad 1 (CP1) were co-modified with specific AU probes and EN-prob1. AuNPs dispensed on the conjugation pad 2 (CP2) were modified with EN-prob2, which were complementary to EN-prob1. Hence, after the T-lines were formed, the secondary conjugates could bind to the AuNPs immobilized on the T-lines, which could enhance the intensity of the T-line and sensitivity of the biosensor. The different ratio of AU probes and EN-prob1 showed different signal enhancement of the biosensor (Figure 4A). When the ratio of specific AU probes to EN-prob1 was 1:1, the signal of the T-line was significantly enhanced. A signal of T-Line of 2.5 nM A-prod was equivalent to 10 nM A-prod on the control biosensor, indicating a four-fold signal enhancement of the control biosensor (Figure 4B).

The preliminary feasibility experiments showed that even without specific SDA primers, positive results still could be obtained in positive sample detection. At first, we thought it was caused by pollution, but the same results were observed in all the subsequent repeated experiments. Finally, we realized that the RNA fragments in the cDNA/RNA hybrid chain generated by the RNase H activity of the AMV enzyme acts as an SDA primer, which initiates the SDA reaction, just like the synthesis of a second strand of a cDNA. Further experiments showed that the signal of the T-line for 20 μL lysis mixture detection without SDA primers was significantly weaker than that with SDA primers, and it was equivalent to the signal of the T-line for 10 μL lysis mixture plus 10 μL lysis buffer detection with SDA primers (Figure 4C). So, the amplification efficiency of the enzyme mixture without SDA primers decreased by less than 50% compared to systems with this primer. And the enzyme mixture with SDA primer was selected as our final solution.

### 3.4. Sensitivity and Specificity of the Biosensor for Different Viruses

**Figure 5:**
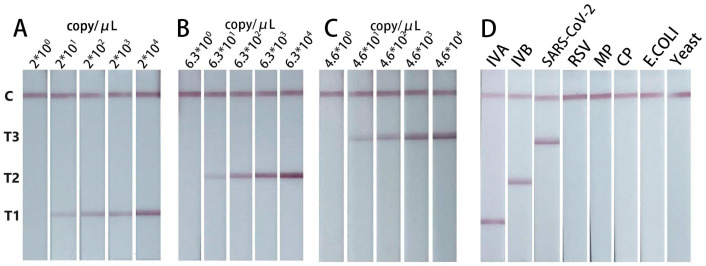
Sensitivity and specificity of the biosensor. Sensitivity for influenza A (**A**), sensitivity for influenza B (**B**) and sensitivity for SARS-CoV-2 (**C**). (**D**) Typical images of biosensor for different viruses. From left to right are influenza A virus (IVA), influenza B virus (IVB), SARS-CoV-2, respiratory syncytial virus (RSV), mycoplasma pneumoniae (MP), chlamydia pneumoniae (MP), *Escherichia coli* (*E. coli*) and yeast.

After optimization of the parameters, the sensitivity of the biosensor was assessed using gradient-diluted samples (inactivated virus or swab elution). The results showed that the LOD of influenza A, influenza B and SARS-CoV-2 was 2 × 10^1^ copy/μL, 6.3 × 10^1^ copy/μL and 4.6 × 10^1^ copy/μL, respectively. The present biosensor also showed good specificity that no test line was observed in the detection of respiratory syncytial virus, mycoplasma pneumoniae, chlamydia pneumoniae, *Escherichia coli* and yeast at 300 copy/μL (Figure 5D).

### 3.5. The Detection of Spiked Samples

**Figure 6:**
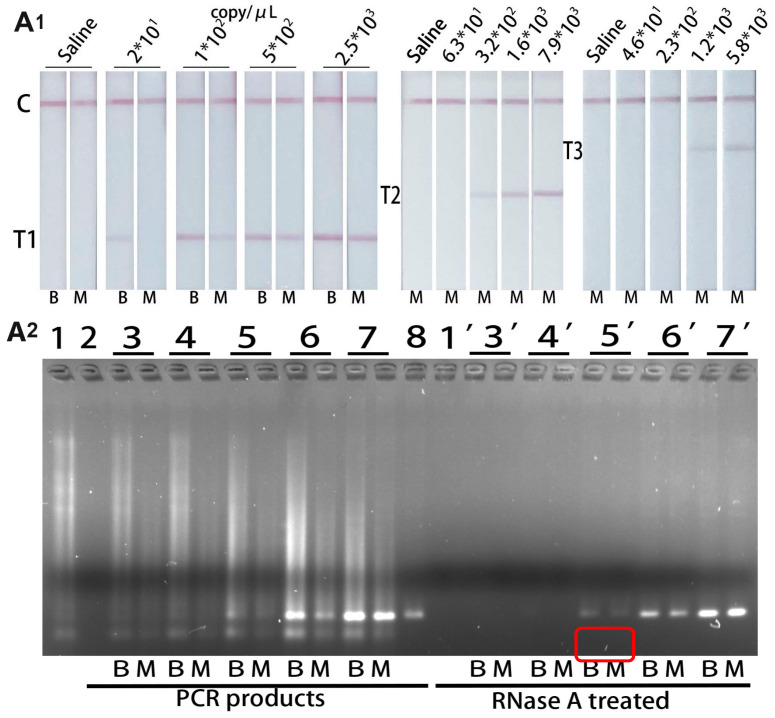
(**A1**) Typical images of the biosensor for virus-spiked samples detection. T1-line was specific for influenza A. T2-line was specific for influenza B. T3-line was specific for SARS-CoV-2. Results of inactivated virus diluted in lysis buffer were marked as “B”. Results of inactivated virus diluted in matrix (spiked sample) were marked as “M”. (**A2**) RT-PCR confirmation of influenza A virus in lysis buffer and spiked samples. 1 & 1′. Carrier RNA, 2. Negative control for PCR, 3 & 3′. Saline, 4 & 4′. 2 × 10^1^ copy/μL influenza A virus. 5 & 5′. 1 × 10^2^ copy/μL influenza A virus, 6 & 6′. 5 × 10^2^ copy/μL and 2.5 × 10^3^ copy/μL influenza A virus, 8. Positive control for PCR. Symbol “ ‘ ” represents that the sample has been treated by RNase A for 30 min. Results of inactivated virus diluted in lysis buffer were marked as ‘B’. Results of inactivated virus diluted in matrix (spiked sample) were marked as “M”.

The nasal swab elution free of the three target viruses was lysed with lysis buffer (without carrier RNA), and the obtained lysis buffer was used as a sample matrix. The results showed that the LOD of influenza A, influenza B and SARS-CoV-2 in spiked samples was 1 × 10^2^ copy/μL, 3.2 × 10^2^ copy/μL and 1.2 × 10^3^ copy/μL, respectively (Figure 6(A1)). RT-PCR confirmation of influenza A virus showed that the LOD of influenza A was 1 × 10^2^ copy/μL in spiked samples (Figure 6(A2)). The LOD for influenza A of this assay was comparable to RT-PCR. So, this assay has good sensitivity in the detection of spiked samples.

**Figure 7:**
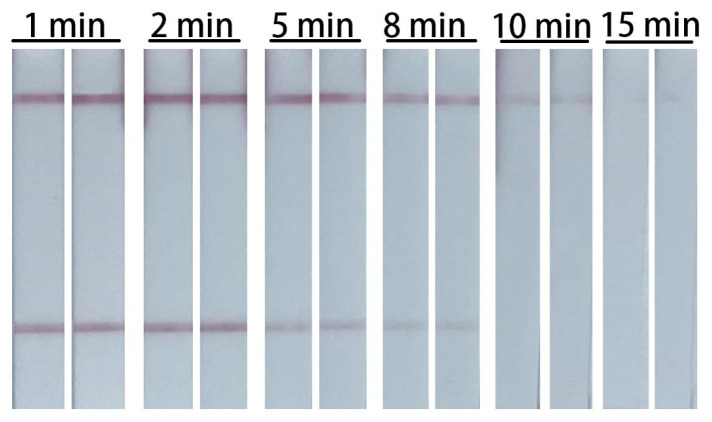
Typical images of the biosensor for the detection of influenza A virus with different lysis time. The concentration of the target RNA for this experiment was 2.5 × 10^3^ copy/μL.

Since RNAs could be degraded by RNase in the lysis buffer (Figure 6(A2)). It is crucial to determine the best lysis time of this assay. The results showed that 2 min lysis resulted the highest intensity of the T-line. The intensity of the T-line decreased dramatically if the lysis time exceeded 5 min, even resulting in false negative results (Figure 7). Therefore, the lysis process should be controlled within 2 min.

**Figure 8:**
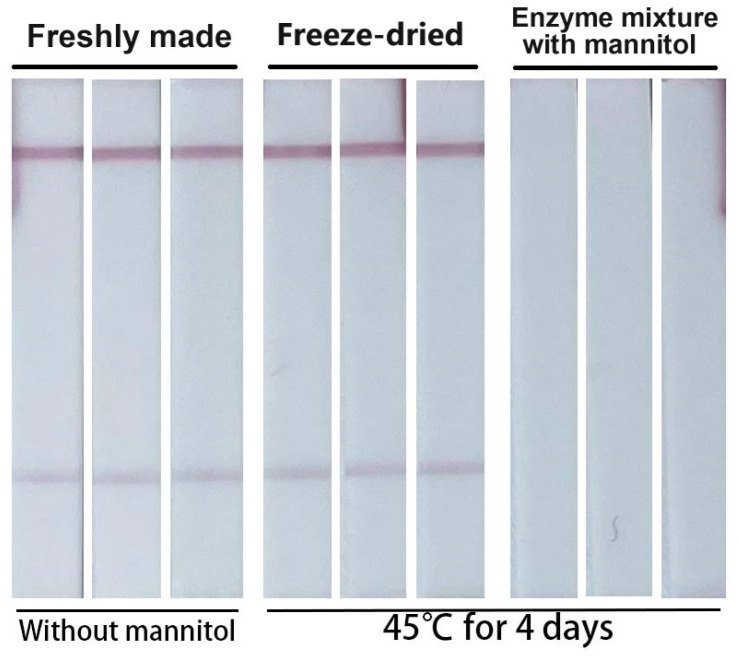
Typical images of the biosensor for the detection of influenza A virus with different enzyme mixture. From left to right are freshly made enzyme mixture, freeze-dried amplification tube (45 °C for 4 days) and the enzyme mixture used for freeze-drying (45 °C for 4 days). The concentration of the target RNA used in this experiment was 5 × 10^2^ copy/μL.

Pre-made enzyme mixtures make this assay more suitable for on-site testing, but before that, we need to evaluate the stability of the freeze-dried enzyme mixture. The freeze-dried amplification tubes and the enzyme mixture used for freeze drying were stored at 45 °C for 4 days. Afterwards, one spiked sample was divided evenly into three parts, and amplified using a freshly made enzyme mixture, 45 °C treated freeze-dried amplification tube and 45 °C treated enzyme mixture. The results showed that performance of the freeze-dried amplification tube was almost the same as that of the freshly made enzyme mixture. While the 45 °C treated enzyme mixture had completely lost its amplification ability (Figure 8). The results indicate that pre-made freeze-dried amplification tubes are easier to store and transport, which makes this assay more suitable for rapid on-site testing.

## 4. Discussion

Various nucleic acid assays for pathogenic microorganism detection have been developed, but these methods had some common shortcomings, especially regarding their complex extraction and amplification procedures [35,36]. Environmental pollution could cause false results, which impedes the development of extraction-independent nucleic acid amplification methods on POCT [37,38]. Since the target sequence in this assay is very short (less than 80 nt) and locates in the middle of target RNA, the enzyme mixture can reverse transcribe and amplify the target sequence immediately after the release of nucleic acid with the protection of carrier RNA, as the excessive carrier RNA can consume the RNase in the system, indirectly protecting and slowing down the degradation of target RNA. Therefore, the biosensor does not require an extraction process. For multiplex target detection, SDA primers can also be omitted to reduce the complexity of the system. The LOD of this biosensor could reach about 20 copy/μL. The high sensitivity and specificity meet the requirements of simultaneous and multiplex on-site detection of pathogens.

The biosensor we developed has the following advantages compared to traditional methods. Firstly, it is easy to operate, which is also the primary concern of all on-site diagnosis. This assay avoids tedious nucleic acid extraction. The integration of RT and SDA reactions into one pre-made ready-to-use amplification tube makes the amplification simpler and consequently reduces the need for complex equipment and skilled operators. Therefore, this method is more suitable for on-site testing. Secondly, the result interpretation is simple and fast. The results can be observed in 5 min by the naked eye. It is faster and more convenient than traditional electrophoresis methods. Thirdly, the specificity of this biosensor is higher than that of traditional nucleic acid detection methods. There are three “checkpoints” for the specificity, the recognition site of the nick enzyme, and the recognition site of primers for RT and SDA, and the probes on AuNPs and the T-line specifically complement the yielded ssDNA. More importantly, the amplified ssDNA cannot be used as a template for further amplification. Therefore, the amplified enzyme mixture will not cause aerosol contamination.

## 5. Conclusions

In conclusion, we developed an on-site multiplex lateral flow biosensor for simultaneous detection of three kinds of viruses. This biosensor avoided the tedious RNA extraction by fast lysis with the aid of carrier RNA. The signal of ssDNAs was further amplified by Au-NPs in the second conjugation pad, which rendered this biosensor highly sensitive. It could be used for the on-site multiplex detection of many other pathogens by changing the primers.

## Figures and Tables

**Table 1:** Oligonucleotide sequences.

Oligonucleotide	Sequence 5′-3′
IVA-RT@	CATTTGAA
IVA-SDA	TCATTGGGATCTTG
IVA-AU2x *	TAATCCACAATTAATCCACAAT—biotin
IVA-Capture2x *	AAAAGACGATCAAAAAGACGATCA
IVA-Control	ACCTGATATTGTGGATACTGATCGTCTTTTTTTCAAATG
IVA-F	CCCTCAAAGCCGAGATCGC
IVA-R	CTGGGCACGGTGAGCGTGAA
IVB-RT	ATCTAATTGTG
IVB-SDA	ACAATGGTGGAT
IVB-AU	CTTCGGGTAATGGTCCA—biotin
IVB-Capture	CATAGGCACTCGGCTCA
1AB-RT	TACTTAAGATTCA
1AB-SDA	TGAGTTATGAGGAT
1AB-AU	Biotin—GAGTTATAGTAGGGATGAC
1AB-Capture2x *	CGTTTTGTATATGCGCGTTTTGTATATGCG
Actin-RT	GCTTGGTGCGAG
Actin-SDA	GTTCTCAGTGGTGG
Actin-AU	GGTCAGCAATACCAGGGAAC—biotin
Actin-Capture	CAGTGATCTCCTTGCTCATA
En-prob 1 ^#^	GTCCTCGCTCACTGGTTTTT—biotin
En-prob 2 ^#^	CCAGTGAGCGAGGACCCAGTGAGCGAGGAC—biotin

Notes: IVA means the probes for influenza A virus, and IVB for influenza B virus, 1AB for SARS-CoV-2 and Actin for β-actin mRNA of rice. * Means repeat the needed sequence one time. ^#^ Signal amplification system probes.

## Data Availability

All the authors are willing to share all the research data of this paper, but no new data were created.
